# Algebraic comparison of metabolic networks, phylogenetic inference, and metabolic innovation

**DOI:** 10.1186/1471-2105-7-67

**Published:** 2006-02-14

**Authors:** Christian V Forst, Christoph Flamm, Ivo L Hofacker, Peter F Stadler

**Affiliations:** 1 Bioscience Division, Los Alamos National Laboratory, Mailstop M888, P.O. Box 1663, Los Alamos, NM 87545, USA; 2 Department of Theoretical Chemistry, University of Vienna, Währingerstraβe 17, A-1090 Wien, Austria; 3 Bioinformatics Group, Department of Computer Science, and Interdisciplinary Center for Bioinformatics, University of Leipzig, Härtelstraβe 16-18, D-04107 Leipzig, Germany; 4 Santa Fe Institute, 1399 Hyde Park Rd., Santa Fe, NM 87501, USA

## Abstract

**Background:**

Comparison of metabolic networks is typically performed based on the organisms' enzyme contents. This approach disregards functional replacements as well as orthologies that are misannotated. Direct comparison of the structure of metabolic networks can circumvent these problems.

**Results:**

Metabolic networks are naturally represented as directed hypergraphs in such a way that metabolites are nodes and enzyme-catalyzed reactions form (hyper)edges. The familiar operations from set algebra (union, intersection, and difference) form a natural basis for both the pairwise comparison of networks and identification of distinct metabolic features of a set of algorithms. We report here on an implementation of this approach and its application to the procaryotes.

**Conclusion:**

We demonstrate that metabolic networks contain valuable phylogenetic information by comparing phylogenies obtained from network comparisons with 16S RNA phylogenies. The algebraic approach to metabolic networks is suitable to study metabolic innovations in two sets of organisms, free living microbes and *Pyrococci*, as well as obligate intracellular pathogens.

## Background

The metabolic networks of a wide variety of organisms, in particular procaryotes, have been reconstructed by means of a combination of genomic annotations with biochemical and physiological data, see e.g. [1]. These networks are compiled in databases, in particular in the KEGG resource [2].

Large scale bacterial phylogenies that are based on single genes are notoriously plagued by gene transfer, gene duplication, gene deletion, and functional replacement of genes. The same holds for various approaches towards utilizing gene content for phylogenetic purposes, discussed e.g. by [3-8]. A recent article by [9] addressed this issue by considering the presence or absence of 64 individual subpathways that were identified based on the COG division [10] of the National Center for Biotechnology Information. A related approach, based on comparison of individual pathways was discussed by [11-13] and [14]. The pathways necessary for such approaches can be derived from a given metabolic network either "by hand" or using automated procedures such as metabolic flux analysis, see e.g. [15-19].

Instead of attempting to first reconstruct individual pathways, we take here a more global view by grounding our analysis in the direct comparison of the metabolic networks. While the application of generic graph distances or similarity measures (see e.g. [20]) is certainly appealing, they cannot be used in a straightforward manner for metabolic networks. The reason is that chemical reaction networks do not have a simple representation as graphs, at least not when metabolites are represented as nodes and reactions as edges. Instead, a metabolic network is naturally described by a directed hypergraph [21], or, equivalently, by a directed bipartite graph, in which metabolites and reactions (or, equivalently, the enzymes that catalyze the reactions), are represented by two different types of vertices. More global comparison of metabolic networks, in terms of various network indices and networks motifs, can be found in [22].

This contribution is organized as follows: In the next section we summarize an algebraic approach to comparison and manipulation of chemical reaction networks that is motivated by set theory. We briefly describe the C library that implements this approach. We then demonstrate that the symmetric difference of two metabolic networks can be used to derive a distance measure that is suitable for reconstructing phylogenetic relationship from metabolic network data. More interestingly, however, the same approach can be used directly to extract those subnetworks of the metabolism that are innovations in the particular subtree of the phylogeny. We illustrate our approach using pathogenic procaryotes as an example.

### The algebra of directed hypergraphs

A metabolic network is defined by its metabolites and the system of reactions that inter-converts them. We denote the set of metabolites by *X*. A chemical reaction *E *can be described as a pair of multisets (*E*^-^, *E*^+^), where *E*^- ^⊆ *X *is the set of educts in the reaction and *E*^+ ^⊆ *X *is the set of reaction products. Slightly more generally, we can replace the multisets by ordinary sets and instead define the multiplicities of product and educt metabolites by means of the stoichiometric coefficients nx,E+
 MathType@MTEF@5@5@+=feaafiart1ev1aaatCvAUfKttLearuWrP9MDH5MBPbIqV92AaeXatLxBI9gBaebbnrfifHhDYfgasaacH8akY=wiFfYdH8Gipec8Eeeu0xXdbba9frFj0=OqFfea0dXdd9vqai=hGuQ8kuc9pgc9s8qqaq=dirpe0xb9q8qiLsFr0=vr0=vr0dc8meaabaqaciaacaGaaeqabaqabeGadaaakeaacqWGUbGBdaqhaaWcbaGaemiEaGNaeiilaWIaemyraueabaGaey4kaScaaaaa@328C@ and nx,E−
 MathType@MTEF@5@5@+=feaafiart1ev1aaatCvAUfKttLearuWrP9MDH5MBPbIqV92AaeXatLxBI9gBaebbnrfifHhDYfgasaacH8akY=wiFfYdH8Gipec8Eeeu0xXdbba9frFj0=OqFfea0dXdd9vqai=hGuQ8kuc9pgc9s8qqaq=dirpe0xb9q8qiLsFr0=vr0=vr0dc8meaabaqaciaacaGaaeqabaqabeGadaaakeaacqWGUbGBdaqhaaWcbaGaemiEaGNaeiilaWIaemyraueabaGaeyOeI0caaaaa@3297@ of the products and educts, respectively. A metabolic network is thus a pair (*X*, ℰ
 MathType@MTEF@5@5@+=feaafiart1ev1aaatCvAUfKttLearuWrP9MDH5MBPbIqV92AaeXatLxBI9gBamrtHrhAL1wy0L2yHvtyaeHbnfgDOvwBHrxAJfwnaebbnrfifHhDYfgasaacH8akY=wiFfYdH8Gipec8Eeeu0xXdbba9frFj0=OqFfea0dXdd9vqai=hGuQ8kuc9pgc9s8qqaq=dirpe0xb9q8qiLsFr0=vr0=vr0dc8meaabaqaciaacaGaaeqabaWaaeGaeaaakeaaimaacqWFWesraaa@3785@) where ℰ
 MathType@MTEF@5@5@+=feaafiart1ev1aaatCvAUfKttLearuWrP9MDH5MBPbIqV92AaeXatLxBI9gBamrtHrhAL1wy0L2yHvtyaeHbnfgDOvwBHrxAJfwnaebbnrfifHhDYfgasaacH8akY=wiFfYdH8Gipec8Eeeu0xXdbba9frFj0=OqFfea0dXdd9vqai=hGuQ8kuc9pgc9s8qqaq=dirpe0xb9q8qiLsFr0=vr0=vr0dc8meaabaqaciaacaGaaeqabaWaaeGaeaaakeaaimaacqWFWesraaa@3785@ is a set of reactions. Such a structure is known as a directed hypergraph M
 MathType@MTEF@5@5@+=feaafiart1ev1aaatCvAUfKttLearuWrP9MDH5MBPbIqV92AaeXatLxBI9gBamrtHrhAL1wy0L2yHvtyaeHbnfgDOvwBHrxAJfwnaebbnrfifHhDYfgasaacH8akY=wiFfYdH8Gipec8Eeeu0xXdbba9frFj0=OqFfea0dXdd9vqai=hGuQ8kuc9pgc9s8qqaq=dirpe0xb9q8qiLsFr0=vr0=vr0dc8meaabaqaciaacaGaaeqabaWaaeGaeaaakeaat0uy0HwzTfgDPnwy2aqeh0uy0HwzTfgDPnwy2aacfaGae8hdW3eaaa@418D@(*X*, ℰ
 MathType@MTEF@5@5@+=feaafiart1ev1aaatCvAUfKttLearuWrP9MDH5MBPbIqV92AaeXatLxBI9gBamrtHrhAL1wy0L2yHvtyaeHbnfgDOvwBHrxAJfwnaebbnrfifHhDYfgasaacH8akY=wiFfYdH8Gipec8Eeeu0xXdbba9frFj0=OqFfea0dXdd9vqai=hGuQ8kuc9pgc9s8qqaq=dirpe0xb9q8qiLsFr0=vr0=vr0dc8meaabaqaciaacaGaaeqabaWaaeGaeaaakeaaimaacqWFWesraaa@3785@), see e.g. [21]. The *stoichiometric matrix ***S **of the network has the entries

SxE=nx,E+−nx,E−     (1)
 MathType@MTEF@5@5@+=feaafiart1ev1aaatCvAUfKttLearuWrP9MDH5MBPbIqV92AaeXatLxBI9gBaebbnrfifHhDYfgasaacH8akY=wiFfYdH8Gipec8Eeeu0xXdbba9frFj0=OqFfea0dXdd9vqai=hGuQ8kuc9pgc9s8qqaq=dirpe0xb9q8qiLsFr0=vr0=vr0dc8meaabaqaciaacaGaaeqabaqabeGadaaakeaaieqacqWFtbWudaWgaaWcbaGaemiEaGNaemyraueabeaakiabg2da9iabd6gaUnaaDaaaleaacqWG4baEcqGGSaalcqWGfbqraeaacqGHRaWkaaGccqGHsislcqWGUbGBdaqhaaWcbaGaemiEaGNaeiilaWIaemyraueabaGaeyOeI0caaOGaaCzcaiaaxMaadaqadaqaaiabigdaXaGaayjkaiaawMcaaaaa@4232@

where *x *∈ *X *is a metabolite, and *E *is a reaction. For completeness, we remark that the set *E*^*c *^= *E*^+ ^∩ *E*^- ^are the catalysts of the reaction *E*. Furthermore, a reaction is autocatalytic if nx,E+
 MathType@MTEF@5@5@+=feaafiart1ev1aaatCvAUfKttLearuWrP9MDH5MBPbIqV92AaeXatLxBI9gBaebbnrfifHhDYfgasaacH8akY=wiFfYdH8Gipec8Eeeu0xXdbba9frFj0=OqFfea0dXdd9vqai=hGuQ8kuc9pgc9s8qqaq=dirpe0xb9q8qiLsFr0=vr0=vr0dc8meaabaqaciaacaGaaeqabaqabeGadaaakeaacqWGUbGBdaqhaaWcbaGaemiEaGNaeiilaWIaemyraueabaGaey4kaScaaaaa@328C@ - nx,E−
 MathType@MTEF@5@5@+=feaafiart1ev1aaatCvAUfKttLearuWrP9MDH5MBPbIqV92AaeXatLxBI9gBaebbnrfifHhDYfgasaacH8akY=wiFfYdH8Gipec8Eeeu0xXdbba9frFj0=OqFfea0dXdd9vqai=hGuQ8kuc9pgc9s8qqaq=dirpe0xb9q8qiLsFr0=vr0=vr0dc8meaabaqaciaacaGaaeqabaqabeGadaaakeaacqWGUbGBdaqhaaWcbaGaemiEaGNaeiilaWIaemyraueabaGaeyOeI0caaaaa@3297@ ≠ 0 for some *x *∈ *E*^*c*^. By abuse of notation we write *E *= *E*^+ ^∪ *E*^- ^for the set of metabolites involved in the reaction *E*. Furthermore, we write suppℰ
 MathType@MTEF@5@5@+=feaafiart1ev1aaatCvAUfKttLearuWrP9MDH5MBPbIqV92AaeXatLxBI9gBamrtHrhAL1wy0L2yHvtyaeHbnfgDOvwBHrxAJfwnaebbnrfifHhDYfgasaacH8akY=wiFfYdH8Gipec8Eeeu0xXdbba9frFj0=OqFfea0dXdd9vqai=hGuQ8kuc9pgc9s8qqaq=dirpe0xb9q8qiLsFr0=vr0=vr0dc8meaabaqaciaacaGaaeqabaWaaeGaeaaakeaaimaacqWFWesraaa@3785@ = ∪{*E*|*E *∈ ℰ
 MathType@MTEF@5@5@+=feaafiart1ev1aaatCvAUfKttLearuWrP9MDH5MBPbIqV92AaeXatLxBI9gBamrtHrhAL1wy0L2yHvtyaeHbnfgDOvwBHrxAJfwnaebbnrfifHhDYfgasaacH8akY=wiFfYdH8Gipec8Eeeu0xXdbba9frFj0=OqFfea0dXdd9vqai=hGuQ8kuc9pgc9s8qqaq=dirpe0xb9q8qiLsFr0=vr0=vr0dc8meaabaqaciaacaGaaeqabaWaaeGaeaaakeaaimaacqWFWesraaa@3785@} for the set metabolites that actually take part in the reactions. We call a network M
 MathType@MTEF@5@5@+=feaafiart1ev1aaatCvAUfKttLearuWrP9MDH5MBPbIqV92AaeXatLxBI9gBamrtHrhAL1wy0L2yHvtyaeHbnfgDOvwBHrxAJfwnaebbnrfifHhDYfgasaacH8akY=wiFfYdH8Gipec8Eeeu0xXdbba9frFj0=OqFfea0dXdd9vqai=hGuQ8kuc9pgc9s8qqaq=dirpe0xb9q8qiLsFr0=vr0=vr0dc8meaabaqaciaacaGaaeqabaWaaeGaeaaakeaat0uy0HwzTfgDPnwy2aqeh0uy0HwzTfgDPnwy2aacfaGae8hdW3eaaa@418D@(*X*, ℰ
 MathType@MTEF@5@5@+=feaafiart1ev1aaatCvAUfKttLearuWrP9MDH5MBPbIqV92AaeXatLxBI9gBamrtHrhAL1wy0L2yHvtyaeHbnfgDOvwBHrxAJfwnaebbnrfifHhDYfgasaacH8akY=wiFfYdH8Gipec8Eeeu0xXdbba9frFj0=OqFfea0dXdd9vqai=hGuQ8kuc9pgc9s8qqaq=dirpe0xb9q8qiLsFr0=vr0=vr0dc8meaabaqaciaacaGaaeqabaWaaeGaeaaakeaaimaacqWFWesraaa@3785@) *clean *if *X *= suppℰ
 MathType@MTEF@5@5@+=feaafiart1ev1aaatCvAUfKttLearuWrP9MDH5MBPbIqV92AaeXatLxBI9gBamrtHrhAL1wy0L2yHvtyaeHbnfgDOvwBHrxAJfwnaebbnrfifHhDYfgasaacH8akY=wiFfYdH8Gipec8Eeeu0xXdbba9frFj0=OqFfea0dXdd9vqai=hGuQ8kuc9pgc9s8qqaq=dirpe0xb9q8qiLsFr0=vr0=vr0dc8meaabaqaciaacaGaaeqabaWaaeGaeaaakeaaimaacqWFWesraaa@3785@ and define the *clean up operator *as M
 MathType@MTEF@5@5@+=feaafiart1ev1aaatCvAUfKttLearuWrP9MDH5MBPbIqV92AaeXatLxBI9gBamrtHrhAL1wy0L2yHvtyaeHbnfgDOvwBHrxAJfwnaebbnrfifHhDYfgasaacH8akY=wiFfYdH8Gipec8Eeeu0xXdbba9frFj0=OqFfea0dXdd9vqai=hGuQ8kuc9pgc9s8qqaq=dirpe0xb9q8qiLsFr0=vr0=vr0dc8meaabaqaciaacaGaaeqabaWaaeGaeaaakeaat0uy0HwzTfgDPnwy2aqeh0uy0HwzTfgDPnwy2aacfaGae8hdW3eaaa@418D@ = (suppℰ
 MathType@MTEF@5@5@+=feaafiart1ev1aaatCvAUfKttLearuWrP9MDH5MBPbIqV92AaeXatLxBI9gBamrtHrhAL1wy0L2yHvtyaeHbnfgDOvwBHrxAJfwnaebbnrfifHhDYfgasaacH8akY=wiFfYdH8Gipec8Eeeu0xXdbba9frFj0=OqFfea0dXdd9vqai=hGuQ8kuc9pgc9s8qqaq=dirpe0xb9q8qiLsFr0=vr0=vr0dc8meaabaqaciaacaGaaeqabaWaaeGaeaaakeaaimaacqWFWesraaa@3785@, ℰ
 MathType@MTEF@5@5@+=feaafiart1ev1aaatCvAUfKttLearuWrP9MDH5MBPbIqV92AaeXatLxBI9gBamrtHrhAL1wy0L2yHvtyaeHbnfgDOvwBHrxAJfwnaebbnrfifHhDYfgasaacH8akY=wiFfYdH8Gipec8Eeeu0xXdbba9frFj0=OqFfea0dXdd9vqai=hGuQ8kuc9pgc9s8qqaq=dirpe0xb9q8qiLsFr0=vr0=vr0dc8meaabaqaciaacaGaaeqabaWaaeGaeaaakeaaimaacqWFWesraaa@3785@). Furthermore, for a given set ℰ
 MathType@MTEF@5@5@+=feaafiart1ev1aaatCvAUfKttLearuWrP9MDH5MBPbIqV92AaeXatLxBI9gBamrtHrhAL1wy0L2yHvtyaeHbnfgDOvwBHrxAJfwnaebbnrfifHhDYfgasaacH8akY=wiFfYdH8Gipec8Eeeu0xXdbba9frFj0=OqFfea0dXdd9vqai=hGuQ8kuc9pgc9s8qqaq=dirpe0xb9q8qiLsFr0=vr0=vr0dc8meaabaqaciaacaGaaeqabaWaaeGaeaaakeaaimaacqWFWesraaa@3785@ of reactions and set *A *metabolites we define

ℰ
 MathType@MTEF@5@5@+=feaafiart1ev1aaatCvAUfKttLearuWrP9MDH5MBPbIqV92AaeXatLxBI9gBamrtHrhAL1wy0L2yHvtyaeHbnfgDOvwBHrxAJfwnaebbnrfifHhDYfgasaacH8akY=wiFfYdH8Gipec8Eeeu0xXdbba9frFj0=OqFfea0dXdd9vqai=hGuQ8kuc9pgc9s8qqaq=dirpe0xb9q8qiLsFr0=vr0=vr0dc8meaabaqaciaacaGaaeqabaWaaeGaeaaakeaaimaacqWFWesraaa@3785@[*A*] = {*E *∈ ℰ
 MathType@MTEF@5@5@+=feaafiart1ev1aaatCvAUfKttLearuWrP9MDH5MBPbIqV92AaeXatLxBI9gBamrtHrhAL1wy0L2yHvtyaeHbnfgDOvwBHrxAJfwnaebbnrfifHhDYfgasaacH8akY=wiFfYdH8Gipec8Eeeu0xXdbba9frFj0=OqFfea0dXdd9vqai=hGuQ8kuc9pgc9s8qqaq=dirpe0xb9q8qiLsFr0=vr0=vr0dc8meaabaqaciaacaGaaeqabaWaaeGaeaaakeaaimaacqWFWesraaa@3785@ | (*E*^+ ^∪ *E*^-^) ⊆ *A*}     (2)

The restriction of a network M
 MathType@MTEF@5@5@+=feaafiart1ev1aaatCvAUfKttLearuWrP9MDH5MBPbIqV92AaeXatLxBI9gBamrtHrhAL1wy0L2yHvtyaeHbnfgDOvwBHrxAJfwnaebbnrfifHhDYfgasaacH8akY=wiFfYdH8Gipec8Eeeu0xXdbba9frFj0=OqFfea0dXdd9vqai=hGuQ8kuc9pgc9s8qqaq=dirpe0xb9q8qiLsFr0=vr0=vr0dc8meaabaqaciaacaGaaeqabaWaaeGaeaaakeaat0uy0HwzTfgDPnwy2aqeh0uy0HwzTfgDPnwy2aacfaGae8hdW3eaaa@418D@(*X*, *E*) to a set *A *of metabolites is defined as the clean network

M
 MathType@MTEF@5@5@+=feaafiart1ev1aaatCvAUfKttLearuWrP9MDH5MBPbIqV92AaeXatLxBI9gBamrtHrhAL1wy0L2yHvtyaeHbnfgDOvwBHrxAJfwnaebbnrfifHhDYfgasaacH8akY=wiFfYdH8Gipec8Eeeu0xXdbba9frFj0=OqFfea0dXdd9vqai=hGuQ8kuc9pgc9s8qqaq=dirpe0xb9q8qiLsFr0=vr0=vr0dc8meaabaqaciaacaGaaeqabaWaaeGaeaaakeaat0uy0HwzTfgDPnwy2aqeh0uy0HwzTfgDPnwy2aacfaGae8hdW3eaaa@418D@[*A*] = (*A*, ℰ
 MathType@MTEF@5@5@+=feaafiart1ev1aaatCvAUfKttLearuWrP9MDH5MBPbIqV92AaeXatLxBI9gBamrtHrhAL1wy0L2yHvtyaeHbnfgDOvwBHrxAJfwnaebbnrfifHhDYfgasaacH8akY=wiFfYdH8Gipec8Eeeu0xXdbba9frFj0=OqFfea0dXdd9vqai=hGuQ8kuc9pgc9s8qqaq=dirpe0xb9q8qiLsFr0=vr0=vr0dc8meaabaqaciaacaGaaeqabaWaaeGaeaaakeaaimaacqWFWesraaa@3785@[*A*]).     (3)

For short we write M
 MathType@MTEF@5@5@+=feaafiart1ev1aaatCvAUfKttLearuWrP9MDH5MBPbIqV92AaeXatLxBI9gBamrtHrhAL1wy0L2yHvtyaeHbnfgDOvwBHrxAJfwnaebbnrfifHhDYfgasaacH8akY=wiFfYdH8Gipec8Eeeu0xXdbba9frFj0=OqFfea0dXdd9vqai=hGuQ8kuc9pgc9s8qqaq=dirpe0xb9q8qiLsFr0=vr0=vr0dc8meaabaqaciaacaGaaeqabaWaaeGaeaaakeaat0uy0HwzTfgDPnwy2aqeh0uy0HwzTfgDPnwy2aacfaGae8hdW3eaaa@418D@ [ℰ
 MathType@MTEF@5@5@+=feaafiart1ev1aaatCvAUfKttLearuWrP9MDH5MBPbIqV92AaeXatLxBI9gBamrtHrhAL1wy0L2yHvtyaeHbnfgDOvwBHrxAJfwnaebbnrfifHhDYfgasaacH8akY=wiFfYdH8Gipec8Eeeu0xXdbba9frFj0=OqFfea0dXdd9vqai=hGuQ8kuc9pgc9s8qqaq=dirpe0xb9q8qiLsFr0=vr0=vr0dc8meaabaqaciaacaGaaeqabaWaaeGaeaaakeaaimaacqWFWesraaa@3785@] = M
 MathType@MTEF@5@5@+=feaafiart1ev1aaatCvAUfKttLearuWrP9MDH5MBPbIqV92AaeXatLxBI9gBamrtHrhAL1wy0L2yHvtyaeHbnfgDOvwBHrxAJfwnaebbnrfifHhDYfgasaacH8akY=wiFfYdH8Gipec8Eeeu0xXdbba9frFj0=OqFfea0dXdd9vqai=hGuQ8kuc9pgc9s8qqaq=dirpe0xb9q8qiLsFr0=vr0=vr0dc8meaabaqaciaacaGaaeqabaWaaeGaeaaakeaat0uy0HwzTfgDPnwy2aqeh0uy0HwzTfgDPnwy2aacfaGae8hdW3eaaa@418D@ [suppℰ
 MathType@MTEF@5@5@+=feaafiart1ev1aaatCvAUfKttLearuWrP9MDH5MBPbIqV92AaeXatLxBI9gBamrtHrhAL1wy0L2yHvtyaeHbnfgDOvwBHrxAJfwnaebbnrfifHhDYfgasaacH8akY=wiFfYdH8Gipec8Eeeu0xXdbba9frFj0=OqFfea0dXdd9vqai=hGuQ8kuc9pgc9s8qqaq=dirpe0xb9q8qiLsFr0=vr0=vr0dc8meaabaqaciaacaGaaeqabaWaaeGaeaaakeaaimaacqWFWesraaa@3785@] for the restriction with respect to a set of reactions. The number of reactions in a network M
 MathType@MTEF@5@5@+=feaafiart1ev1aaatCvAUfKttLearuWrP9MDH5MBPbIqV92AaeXatLxBI9gBamrtHrhAL1wy0L2yHvtyaeHbnfgDOvwBHrxAJfwnaebbnrfifHhDYfgasaacH8akY=wiFfYdH8Gipec8Eeeu0xXdbba9frFj0=OqFfea0dXdd9vqai=hGuQ8kuc9pgc9s8qqaq=dirpe0xb9q8qiLsFr0=vr0=vr0dc8meaabaqaciaacaGaaeqabaWaaeGaeaaakeaat0uy0HwzTfgDPnwy2aqeh0uy0HwzTfgDPnwy2aacfaGae8hdW3eaaa@418D@ will be denoted by ||M
 MathType@MTEF@5@5@+=feaafiart1ev1aaatCvAUfKttLearuWrP9MDH5MBPbIqV92AaeXatLxBI9gBamrtHrhAL1wy0L2yHvtyaeHbnfgDOvwBHrxAJfwnaebbnrfifHhDYfgasaacH8akY=wiFfYdH8Gipec8Eeeu0xXdbba9frFj0=OqFfea0dXdd9vqai=hGuQ8kuc9pgc9s8qqaq=dirpe0xb9q8qiLsFr0=vr0=vr0dc8meaabaqaciaacaGaaeqabaWaaeGaeaaakeaat0uy0HwzTfgDPnwy2aqeh0uy0HwzTfgDPnwy2aacfaGae8hdW3eaaa@418D@||.

**Figure 1:**
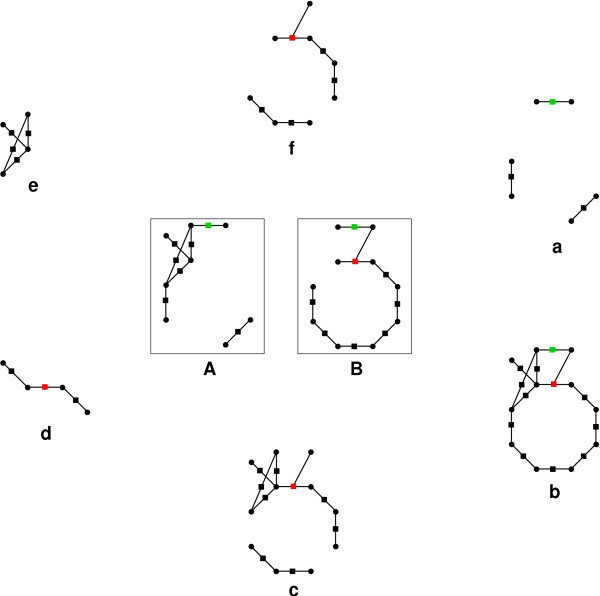
Graphical representation of the basic binary operations of the network algebra. Diagrams (A) and (B) summarize the citric-acid cycle of *P. horikoshii *and *H. pylori *[31]. Hypergraphs can always be drawn as bipartite graphs with one class of vertices representing the hypergraph vertices (chemical species, ●), while the other class of vertices encodes the hyperedges (chemical reactions, ■). Each reaction is connected by (directed) arrows from its educts and to its products. For clarity of presentation we have omitted the direction of the arrows (most reactions are reversible) as well as small molecules such as *CO*_2 _and *H*_2_*O *here. Furthermore, two reactions are marked in color, namely the ones catalyzed by citrate synthase in red, and pyruvate dehydrogenase in green. The results of the basic operations are as follows: (a) Intersection *A *∩ *B*; (b) Union *A *∪ *B*; (c) Symmetric Difference *A *△ *B*; (d) Strict Symmetric Difference *A *◊ *B*; (e) Difference *A *\ *B*; (f) Difference *B *\ *A*.

In order to compare networks in a systematic way, we need to be able to determine the differences and the commonalities of two networks. Inspired by the usual algebra of sets, which is based on the operations "union", "intersection", and "difference", we introduce analogous mathematical constructions for chemical reaction networks. Fig. 1 illustrates the basic operations of this "network algebra" which are formally defined below.

In the following let ' (*X'*, ε') and '' (*X''*, ε'') be two networks. Of course we have ' = ''iff *X'* = *X''* and ε' = ε''. The empty network will be denoted by ∅.

#### Union

The union M
 MathType@MTEF@5@5@+=feaafiart1ev1aaatCvAUfKttLearuWrP9MDH5MBPbIqV92AaeXatLxBI9gBamrtHrhAL1wy0L2yHvtyaeHbnfgDOvwBHrxAJfwnaebbnrfifHhDYfgasaacH8akY=wiFfYdH8Gipec8Eeeu0xXdbba9frFj0=OqFfea0dXdd9vqai=hGuQ8kuc9pgc9s8qqaq=dirpe0xb9q8qiLsFr0=vr0=vr0dc8meaabaqaciaacaGaaeqabaWaaeGaeaaakeaat0uy0HwzTfgDPnwy2aqeh0uy0HwzTfgDPnwy2aacfaGae8hdW3eaaa@418D@ = M′
 MathType@MTEF@5@5@+=feaafiart1ev1aaatCvAUfKttLearuWrP9MDH5MBPbIqV92AaeXatLxBI9gBamrtHrhAL1wy0L2yHvtyaeHbnfgDOvwBHrxAJfwnaebbnrfifHhDYfgasaacH8akY=wiFfYdH8Gipec8Eeeu0xXdbba9frFj0=OqFfea0dXdd9vqai=hGuQ8kuc9pgc9s8qqaq=dirpe0xb9q8qiLsFr0=vr0=vr0dc8meaabaqaciaacaGaaeqabaWaaeGaeaaakeaat0uy0HwzTfgDPnwy2aqeh0uy0HwzTfgDPnwy2aacfaGaf8hdW3Kbauaaaaa@4199@ ∪ M″
 MathType@MTEF@5@5@+=feaafiart1ev1aaatCvAUfKttLearuWrP9MDH5MBPbIqV92AaeXatLxBI9gBamrtHrhAL1wy0L2yHvtyaeHbnfgDOvwBHrxAJfwnaebbnrfifHhDYfgasaacH8akY=wiFfYdH8Gipec8Eeeu0xXdbba9frFj0=OqFfea0dXdd9vqai=hGuQ8kuc9pgc9s8qqaq=dirpe0xb9q8qiLsFr0=vr0=vr0dc8meaabaqaciaacaGaaeqabaWaaeGaeaaakeaat0uy0HwzTfgDPnwy2aqeh0uy0HwzTfgDPnwy2aacfaGaf8hdW3Kbayaaaaa@419A@ is defined as the network (*X' *∪ *X"*, ℰ′
 MathType@MTEF@5@5@+=feaafiart1ev1aaatCvAUfKttLearuWrP9MDH5MBPbIqV92AaeXatLxBI9gBamrtHrhAL1wy0L2yHvtyaeHbnfgDOvwBHrxAJfwnaebbnrfifHhDYfgasaacH8akY=wiFfYdH8Gipec8Eeeu0xXdbba9frFj0=OqFfea0dXdd9vqai=hGuQ8kuc9pgc9s8qqaq=dirpe0xb9q8qiLsFr0=vr0=vr0dc8meaabaqaciaacaGaaeqabaWaaeGaeaaakeaaimaacuWFWesrgaqbaaaa@3791@ ∪ ℰ″
 MathType@MTEF@5@5@+=feaafiart1ev1aaatCvAUfKttLearuWrP9MDH5MBPbIqV92AaeXatLxBI9gBamrtHrhAL1wy0L2yHvtyaeHbnfgDOvwBHrxAJfwnaebbnrfifHhDYfgasaacH8akY=wiFfYdH8Gipec8Eeeu0xXdbba9frFj0=OqFfea0dXdd9vqai=hGuQ8kuc9pgc9s8qqaq=dirpe0xb9q8qiLsFr0=vr0=vr0dc8meaabaqaciaacaGaaeqabaWaaeGaeaaakeaaimaacuWFWesrgaGbaaaa@3792@). Note that M
 MathType@MTEF@5@5@+=feaafiart1ev1aaatCvAUfKttLearuWrP9MDH5MBPbIqV92AaeXatLxBI9gBamrtHrhAL1wy0L2yHvtyaeHbnfgDOvwBHrxAJfwnaebbnrfifHhDYfgasaacH8akY=wiFfYdH8Gipec8Eeeu0xXdbba9frFj0=OqFfea0dXdd9vqai=hGuQ8kuc9pgc9s8qqaq=dirpe0xb9q8qiLsFr0=vr0=vr0dc8meaabaqaciaacaGaaeqabaWaaeGaeaaakeaat0uy0HwzTfgDPnwy2aqeh0uy0HwzTfgDPnwy2aacfaGae8hdW3eaaa@418D@ is clean if both M′
 MathType@MTEF@5@5@+=feaafiart1ev1aaatCvAUfKttLearuWrP9MDH5MBPbIqV92AaeXatLxBI9gBamrtHrhAL1wy0L2yHvtyaeHbnfgDOvwBHrxAJfwnaebbnrfifHhDYfgasaacH8akY=wiFfYdH8Gipec8Eeeu0xXdbba9frFj0=OqFfea0dXdd9vqai=hGuQ8kuc9pgc9s8qqaq=dirpe0xb9q8qiLsFr0=vr0=vr0dc8meaabaqaciaacaGaaeqabaWaaeGaeaaakeaat0uy0HwzTfgDPnwy2aqeh0uy0HwzTfgDPnwy2aacfaGaf8hdW3Kbauaaaaa@4199@ and M″
 MathType@MTEF@5@5@+=feaafiart1ev1aaatCvAUfKttLearuWrP9MDH5MBPbIqV92AaeXatLxBI9gBamrtHrhAL1wy0L2yHvtyaeHbnfgDOvwBHrxAJfwnaebbnrfifHhDYfgasaacH8akY=wiFfYdH8Gipec8Eeeu0xXdbba9frFj0=OqFfea0dXdd9vqai=hGuQ8kuc9pgc9s8qqaq=dirpe0xb9q8qiLsFr0=vr0=vr0dc8meaabaqaciaacaGaaeqabaWaaeGaeaaakeaat0uy0HwzTfgDPnwy2aqeh0uy0HwzTfgDPnwy2aacfaGaf8hdW3Kbayaaaaa@419A@ are clean.

#### Intersection

The intersection M
 MathType@MTEF@5@5@+=feaafiart1ev1aaatCvAUfKttLearuWrP9MDH5MBPbIqV92AaeXatLxBI9gBamrtHrhAL1wy0L2yHvtyaeHbnfgDOvwBHrxAJfwnaebbnrfifHhDYfgasaacH8akY=wiFfYdH8Gipec8Eeeu0xXdbba9frFj0=OqFfea0dXdd9vqai=hGuQ8kuc9pgc9s8qqaq=dirpe0xb9q8qiLsFr0=vr0=vr0dc8meaabaqaciaacaGaaeqabaWaaeGaeaaakeaat0uy0HwzTfgDPnwy2aqeh0uy0HwzTfgDPnwy2aacfaGae8hdW3eaaa@418D@ = M′
 MathType@MTEF@5@5@+=feaafiart1ev1aaatCvAUfKttLearuWrP9MDH5MBPbIqV92AaeXatLxBI9gBamrtHrhAL1wy0L2yHvtyaeHbnfgDOvwBHrxAJfwnaebbnrfifHhDYfgasaacH8akY=wiFfYdH8Gipec8Eeeu0xXdbba9frFj0=OqFfea0dXdd9vqai=hGuQ8kuc9pgc9s8qqaq=dirpe0xb9q8qiLsFr0=vr0=vr0dc8meaabaqaciaacaGaaeqabaWaaeGaeaaakeaat0uy0HwzTfgDPnwy2aqeh0uy0HwzTfgDPnwy2aacfaGaf8hdW3Kbauaaaaa@4199@ ∩ M″
 MathType@MTEF@5@5@+=feaafiart1ev1aaatCvAUfKttLearuWrP9MDH5MBPbIqV92AaeXatLxBI9gBamrtHrhAL1wy0L2yHvtyaeHbnfgDOvwBHrxAJfwnaebbnrfifHhDYfgasaacH8akY=wiFfYdH8Gipec8Eeeu0xXdbba9frFj0=OqFfea0dXdd9vqai=hGuQ8kuc9pgc9s8qqaq=dirpe0xb9q8qiLsFr0=vr0=vr0dc8meaabaqaciaacaGaaeqabaWaaeGaeaaakeaat0uy0HwzTfgDPnwy2aqeh0uy0HwzTfgDPnwy2aacfaGaf8hdW3Kbayaaaaa@419A@ is defined as the clean network

M
 MathType@MTEF@5@5@+=feaafiart1ev1aaatCvAUfKttLearuWrP9MDH5MBPbIqV92AaeXatLxBI9gBamrtHrhAL1wy0L2yHvtyaeHbnfgDOvwBHrxAJfwnaebbnrfifHhDYfgasaacH8akY=wiFfYdH8Gipec8Eeeu0xXdbba9frFj0=OqFfea0dXdd9vqai=hGuQ8kuc9pgc9s8qqaq=dirpe0xb9q8qiLsFr0=vr0=vr0dc8meaabaqaciaacaGaaeqabaWaaeGaeaaakeaat0uy0HwzTfgDPnwy2aqeh0uy0HwzTfgDPnwy2aacfaGae8hdW3eaaa@418D@ = (*X' *∩ *X"*, ℰ′
 MathType@MTEF@5@5@+=feaafiart1ev1aaatCvAUfKttLearuWrP9MDH5MBPbIqV92AaeXatLxBI9gBamrtHrhAL1wy0L2yHvtyaeHbnfgDOvwBHrxAJfwnaebbnrfifHhDYfgasaacH8akY=wiFfYdH8Gipec8Eeeu0xXdbba9frFj0=OqFfea0dXdd9vqai=hGuQ8kuc9pgc9s8qqaq=dirpe0xb9q8qiLsFr0=vr0=vr0dc8meaabaqaciaacaGaaeqabaWaaeGaeaaakeaaimaacuWFWesrgaqbaaaa@3791@ ∩ ℰ″
 MathType@MTEF@5@5@+=feaafiart1ev1aaatCvAUfKttLearuWrP9MDH5MBPbIqV92AaeXatLxBI9gBamrtHrhAL1wy0L2yHvtyaeHbnfgDOvwBHrxAJfwnaebbnrfifHhDYfgasaacH8akY=wiFfYdH8Gipec8Eeeu0xXdbba9frFj0=OqFfea0dXdd9vqai=hGuQ8kuc9pgc9s8qqaq=dirpe0xb9q8qiLsFr0=vr0=vr0dc8meaabaqaciaacaGaaeqabaWaaeGaeaaakeaaimaacuWFWesrgaGbaaaa@3792@)     (4)

Note that (ℰ′
 MathType@MTEF@5@5@+=feaafiart1ev1aaatCvAUfKttLearuWrP9MDH5MBPbIqV92AaeXatLxBI9gBamrtHrhAL1wy0L2yHvtyaeHbnfgDOvwBHrxAJfwnaebbnrfifHhDYfgasaacH8akY=wiFfYdH8Gipec8Eeeu0xXdbba9frFj0=OqFfea0dXdd9vqai=hGuQ8kuc9pgc9s8qqaq=dirpe0xb9q8qiLsFr0=vr0=vr0dc8meaabaqaciaacaGaaeqabaWaaeGaeaaakeaaimaacuWFWesrgaqbaaaa@3791@ ∩ ℰ″
 MathType@MTEF@5@5@+=feaafiart1ev1aaatCvAUfKttLearuWrP9MDH5MBPbIqV92AaeXatLxBI9gBamrtHrhAL1wy0L2yHvtyaeHbnfgDOvwBHrxAJfwnaebbnrfifHhDYfgasaacH8akY=wiFfYdH8Gipec8Eeeu0xXdbba9frFj0=OqFfea0dXdd9vqai=hGuQ8kuc9pgc9s8qqaq=dirpe0xb9q8qiLsFr0=vr0=vr0dc8meaabaqaciaacaGaaeqabaWaaeGaeaaakeaaimaacuWFWesrgaGbaaaa@3792@) [*X' *∩ *X"*] = ℰ′
 MathType@MTEF@5@5@+=feaafiart1ev1aaatCvAUfKttLearuWrP9MDH5MBPbIqV92AaeXatLxBI9gBamrtHrhAL1wy0L2yHvtyaeHbnfgDOvwBHrxAJfwnaebbnrfifHhDYfgasaacH8akY=wiFfYdH8Gipec8Eeeu0xXdbba9frFj0=OqFfea0dXdd9vqai=hGuQ8kuc9pgc9s8qqaq=dirpe0xb9q8qiLsFr0=vr0=vr0dc8meaabaqaciaacaGaaeqabaWaaeGaeaaakeaaimaacuWFWesrgaqbaaaa@3791@ ∩ ℰ″
 MathType@MTEF@5@5@+=feaafiart1ev1aaatCvAUfKttLearuWrP9MDH5MBPbIqV92AaeXatLxBI9gBamrtHrhAL1wy0L2yHvtyaeHbnfgDOvwBHrxAJfwnaebbnrfifHhDYfgasaacH8akY=wiFfYdH8Gipec8Eeeu0xXdbba9frFj0=OqFfea0dXdd9vqai=hGuQ8kuc9pgc9s8qqaq=dirpe0xb9q8qiLsFr0=vr0=vr0dc8meaabaqaciaacaGaaeqabaWaaeGaeaaakeaaimaacuWFWesrgaGbaaaa@3792@.

#### Difference

The *difference *M
 MathType@MTEF@5@5@+=feaafiart1ev1aaatCvAUfKttLearuWrP9MDH5MBPbIqV92AaeXatLxBI9gBamrtHrhAL1wy0L2yHvtyaeHbnfgDOvwBHrxAJfwnaebbnrfifHhDYfgasaacH8akY=wiFfYdH8Gipec8Eeeu0xXdbba9frFj0=OqFfea0dXdd9vqai=hGuQ8kuc9pgc9s8qqaq=dirpe0xb9q8qiLsFr0=vr0=vr0dc8meaabaqaciaacaGaaeqabaWaaeGaeaaakeaat0uy0HwzTfgDPnwy2aqeh0uy0HwzTfgDPnwy2aacfaGae8hdW3eaaa@418D@ = M′
 MathType@MTEF@5@5@+=feaafiart1ev1aaatCvAUfKttLearuWrP9MDH5MBPbIqV92AaeXatLxBI9gBamrtHrhAL1wy0L2yHvtyaeHbnfgDOvwBHrxAJfwnaebbnrfifHhDYfgasaacH8akY=wiFfYdH8Gipec8Eeeu0xXdbba9frFj0=OqFfea0dXdd9vqai=hGuQ8kuc9pgc9s8qqaq=dirpe0xb9q8qiLsFr0=vr0=vr0dc8meaabaqaciaacaGaaeqabaWaaeGaeaaakeaat0uy0HwzTfgDPnwy2aqeh0uy0HwzTfgDPnwy2aacfaGaf8hdW3Kbauaaaaa@4199@\M″
 MathType@MTEF@5@5@+=feaafiart1ev1aaatCvAUfKttLearuWrP9MDH5MBPbIqV92AaeXatLxBI9gBamrtHrhAL1wy0L2yHvtyaeHbnfgDOvwBHrxAJfwnaebbnrfifHhDYfgasaacH8akY=wiFfYdH8Gipec8Eeeu0xXdbba9frFj0=OqFfea0dXdd9vqai=hGuQ8kuc9pgc9s8qqaq=dirpe0xb9q8qiLsFr0=vr0=vr0dc8meaabaqaciaacaGaaeqabaWaaeGaeaaakeaat0uy0HwzTfgDPnwy2aqeh0uy0HwzTfgDPnwy2aacfaGaf8hdW3Kbayaaaaa@419A@ is defined as the clean network

M
 MathType@MTEF@5@5@+=feaafiart1ev1aaatCvAUfKttLearuWrP9MDH5MBPbIqV92AaeXatLxBI9gBamrtHrhAL1wy0L2yHvtyaeHbnfgDOvwBHrxAJfwnaebbnrfifHhDYfgasaacH8akY=wiFfYdH8Gipec8Eeeu0xXdbba9frFj0=OqFfea0dXdd9vqai=hGuQ8kuc9pgc9s8qqaq=dirpe0xb9q8qiLsFr0=vr0=vr0dc8meaabaqaciaacaGaaeqabaWaaeGaeaaakeaat0uy0HwzTfgDPnwy2aqeh0uy0HwzTfgDPnwy2aacfaGae8hdW3eaaa@418D@ = (supp(ℰ′
 MathType@MTEF@5@5@+=feaafiart1ev1aaatCvAUfKttLearuWrP9MDH5MBPbIqV92AaeXatLxBI9gBamrtHrhAL1wy0L2yHvtyaeHbnfgDOvwBHrxAJfwnaebbnrfifHhDYfgasaacH8akY=wiFfYdH8Gipec8Eeeu0xXdbba9frFj0=OqFfea0dXdd9vqai=hGuQ8kuc9pgc9s8qqaq=dirpe0xb9q8qiLsFr0=vr0=vr0dc8meaabaqaciaacaGaaeqabaWaaeGaeaaakeaaimaacuWFWesrgaqbaaaa@3791@\ℰ″
 MathType@MTEF@5@5@+=feaafiart1ev1aaatCvAUfKttLearuWrP9MDH5MBPbIqV92AaeXatLxBI9gBamrtHrhAL1wy0L2yHvtyaeHbnfgDOvwBHrxAJfwnaebbnrfifHhDYfgasaacH8akY=wiFfYdH8Gipec8Eeeu0xXdbba9frFj0=OqFfea0dXdd9vqai=hGuQ8kuc9pgc9s8qqaq=dirpe0xb9q8qiLsFr0=vr0=vr0dc8meaabaqaciaacaGaaeqabaWaaeGaeaaakeaaimaacuWFWesrgaGbaaaa@3792@), ℰ′
 MathType@MTEF@5@5@+=feaafiart1ev1aaatCvAUfKttLearuWrP9MDH5MBPbIqV92AaeXatLxBI9gBamrtHrhAL1wy0L2yHvtyaeHbnfgDOvwBHrxAJfwnaebbnrfifHhDYfgasaacH8akY=wiFfYdH8Gipec8Eeeu0xXdbba9frFj0=OqFfea0dXdd9vqai=hGuQ8kuc9pgc9s8qqaq=dirpe0xb9q8qiLsFr0=vr0=vr0dc8meaabaqaciaacaGaaeqabaWaaeGaeaaakeaaimaacuWFWesrgaqbaaaa@3791@\ℰ″
 MathType@MTEF@5@5@+=feaafiart1ev1aaatCvAUfKttLearuWrP9MDH5MBPbIqV92AaeXatLxBI9gBamrtHrhAL1wy0L2yHvtyaeHbnfgDOvwBHrxAJfwnaebbnrfifHhDYfgasaacH8akY=wiFfYdH8Gipec8Eeeu0xXdbba9frFj0=OqFfea0dXdd9vqai=hGuQ8kuc9pgc9s8qqaq=dirpe0xb9q8qiLsFr0=vr0=vr0dc8meaabaqaciaacaGaaeqabaWaaeGaeaaakeaaimaacuWFWesrgaGbaaaa@3792@)     (5)

The difference network contains all reactions occurring in M′
 MathType@MTEF@5@5@+=feaafiart1ev1aaatCvAUfKttLearuWrP9MDH5MBPbIqV92AaeXatLxBI9gBamrtHrhAL1wy0L2yHvtyaeHbnfgDOvwBHrxAJfwnaebbnrfifHhDYfgasaacH8akY=wiFfYdH8Gipec8Eeeu0xXdbba9frFj0=OqFfea0dXdd9vqai=hGuQ8kuc9pgc9s8qqaq=dirpe0xb9q8qiLsFr0=vr0=vr0dc8meaabaqaciaacaGaaeqabaWaaeGaeaaakeaat0uy0HwzTfgDPnwy2aqeh0uy0HwzTfgDPnwy2aacfaGaf8hdW3Kbauaaaaa@4199@ but not M″
 MathType@MTEF@5@5@+=feaafiart1ev1aaatCvAUfKttLearuWrP9MDH5MBPbIqV92AaeXatLxBI9gBamrtHrhAL1wy0L2yHvtyaeHbnfgDOvwBHrxAJfwnaebbnrfifHhDYfgasaacH8akY=wiFfYdH8Gipec8Eeeu0xXdbba9frFj0=OqFfea0dXdd9vqai=hGuQ8kuc9pgc9s8qqaq=dirpe0xb9q8qiLsFr0=vr0=vr0dc8meaabaqaciaacaGaaeqabaWaaeGaeaaakeaat0uy0HwzTfgDPnwy2aqeh0uy0HwzTfgDPnwy2aacfaGaf8hdW3Kbayaaaaa@419A@, and all metabolites occurring in the remaining reactions.

The *strict difference *M
 MathType@MTEF@5@5@+=feaafiart1ev1aaatCvAUfKttLearuWrP9MDH5MBPbIqV92AaeXatLxBI9gBamrtHrhAL1wy0L2yHvtyaeHbnfgDOvwBHrxAJfwnaebbnrfifHhDYfgasaacH8akY=wiFfYdH8Gipec8Eeeu0xXdbba9frFj0=OqFfea0dXdd9vqai=hGuQ8kuc9pgc9s8qqaq=dirpe0xb9q8qiLsFr0=vr0=vr0dc8meaabaqaciaacaGaaeqabaWaaeGaeaaakeaat0uy0HwzTfgDPnwy2aqeh0uy0HwzTfgDPnwy2aacfaGae8hdW3eaaa@418D@ = M′
 MathType@MTEF@5@5@+=feaafiart1ev1aaatCvAUfKttLearuWrP9MDH5MBPbIqV92AaeXatLxBI9gBamrtHrhAL1wy0L2yHvtyaeHbnfgDOvwBHrxAJfwnaebbnrfifHhDYfgasaacH8akY=wiFfYdH8Gipec8Eeeu0xXdbba9frFj0=OqFfea0dXdd9vqai=hGuQ8kuc9pgc9s8qqaq=dirpe0xb9q8qiLsFr0=vr0=vr0dc8meaabaqaciaacaGaaeqabaWaaeGaeaaakeaat0uy0HwzTfgDPnwy2aqeh0uy0HwzTfgDPnwy2aacfaGaf8hdW3Kbauaaaaa@4199@\\M″
 MathType@MTEF@5@5@+=feaafiart1ev1aaatCvAUfKttLearuWrP9MDH5MBPbIqV92AaeXatLxBI9gBamrtHrhAL1wy0L2yHvtyaeHbnfgDOvwBHrxAJfwnaebbnrfifHhDYfgasaacH8akY=wiFfYdH8Gipec8Eeeu0xXdbba9frFj0=OqFfea0dXdd9vqai=hGuQ8kuc9pgc9s8qqaq=dirpe0xb9q8qiLsFr0=vr0=vr0dc8meaabaqaciaacaGaaeqabaWaaeGaeaaakeaat0uy0HwzTfgDPnwy2aqeh0uy0HwzTfgDPnwy2aacfaGaf8hdW3Kbayaaaaa@419A@ is the clean network

M
 MathType@MTEF@5@5@+=feaafiart1ev1aaatCvAUfKttLearuWrP9MDH5MBPbIqV92AaeXatLxBI9gBamrtHrhAL1wy0L2yHvtyaeHbnfgDOvwBHrxAJfwnaebbnrfifHhDYfgasaacH8akY=wiFfYdH8Gipec8Eeeu0xXdbba9frFj0=OqFfea0dXdd9vqai=hGuQ8kuc9pgc9s8qqaq=dirpe0xb9q8qiLsFr0=vr0=vr0dc8meaabaqaciaacaGaaeqabaWaaeGaeaaakeaat0uy0HwzTfgDPnwy2aqeh0uy0HwzTfgDPnwy2aacfaGae8hdW3eaaa@418D@ = (*X'*\*X"*, (ℰ′
 MathType@MTEF@5@5@+=feaafiart1ev1aaatCvAUfKttLearuWrP9MDH5MBPbIqV92AaeXatLxBI9gBamrtHrhAL1wy0L2yHvtyaeHbnfgDOvwBHrxAJfwnaebbnrfifHhDYfgasaacH8akY=wiFfYdH8Gipec8Eeeu0xXdbba9frFj0=OqFfea0dXdd9vqai=hGuQ8kuc9pgc9s8qqaq=dirpe0xb9q8qiLsFr0=vr0=vr0dc8meaabaqaciaacaGaaeqabaWaaeGaeaaakeaaimaacuWFWesrgaqbaaaa@3791@\ℰ″
 MathType@MTEF@5@5@+=feaafiart1ev1aaatCvAUfKttLearuWrP9MDH5MBPbIqV92AaeXatLxBI9gBamrtHrhAL1wy0L2yHvtyaeHbnfgDOvwBHrxAJfwnaebbnrfifHhDYfgasaacH8akY=wiFfYdH8Gipec8Eeeu0xXdbba9frFj0=OqFfea0dXdd9vqai=hGuQ8kuc9pgc9s8qqaq=dirpe0xb9q8qiLsFr0=vr0=vr0dc8meaabaqaciaacaGaaeqabaWaaeGaeaaakeaaimaacuWFWesrgaGbaaaa@3792@), [*X'*\*X"*])     (6)

The new network contains only those metabolites occurring in M′
 MathType@MTEF@5@5@+=feaafiart1ev1aaatCvAUfKttLearuWrP9MDH5MBPbIqV92AaeXatLxBI9gBamrtHrhAL1wy0L2yHvtyaeHbnfgDOvwBHrxAJfwnaebbnrfifHhDYfgasaacH8akY=wiFfYdH8Gipec8Eeeu0xXdbba9frFj0=OqFfea0dXdd9vqai=hGuQ8kuc9pgc9s8qqaq=dirpe0xb9q8qiLsFr0=vr0=vr0dc8meaabaqaciaacaGaaeqabaWaaeGaeaaakeaat0uy0HwzTfgDPnwy2aqeh0uy0HwzTfgDPnwy2aacfaGaf8hdW3Kbauaaaaa@4199@ but not M″
 MathType@MTEF@5@5@+=feaafiart1ev1aaatCvAUfKttLearuWrP9MDH5MBPbIqV92AaeXatLxBI9gBamrtHrhAL1wy0L2yHvtyaeHbnfgDOvwBHrxAJfwnaebbnrfifHhDYfgasaacH8akY=wiFfYdH8Gipec8Eeeu0xXdbba9frFj0=OqFfea0dXdd9vqai=hGuQ8kuc9pgc9s8qqaq=dirpe0xb9q8qiLsFr0=vr0=vr0dc8meaabaqaciaacaGaaeqabaWaaeGaeaaakeaat0uy0HwzTfgDPnwy2aqeh0uy0HwzTfgDPnwy2aacfaGaf8hdW3Kbayaaaaa@419A@, and only those reactions from M′
 MathType@MTEF@5@5@+=feaafiart1ev1aaatCvAUfKttLearuWrP9MDH5MBPbIqV92AaeXatLxBI9gBamrtHrhAL1wy0L2yHvtyaeHbnfgDOvwBHrxAJfwnaebbnrfifHhDYfgasaacH8akY=wiFfYdH8Gipec8Eeeu0xXdbba9frFj0=OqFfea0dXdd9vqai=hGuQ8kuc9pgc9s8qqaq=dirpe0xb9q8qiLsFr0=vr0=vr0dc8meaabaqaciaacaGaaeqabaWaaeGaeaaakeaat0uy0HwzTfgDPnwy2aqeh0uy0HwzTfgDPnwy2aacfaGaf8hdW3Kbauaaaaa@4199@ that can be performed with the remaining metabolites. Thus, we have ||M′
 MathType@MTEF@5@5@+=feaafiart1ev1aaatCvAUfKttLearuWrP9MDH5MBPbIqV92AaeXatLxBI9gBamrtHrhAL1wy0L2yHvtyaeHbnfgDOvwBHrxAJfwnaebbnrfifHhDYfgasaacH8akY=wiFfYdH8Gipec8Eeeu0xXdbba9frFj0=OqFfea0dXdd9vqai=hGuQ8kuc9pgc9s8qqaq=dirpe0xb9q8qiLsFr0=vr0=vr0dc8meaabaqaciaacaGaaeqabaWaaeGaeaaakeaat0uy0HwzTfgDPnwy2aqeh0uy0HwzTfgDPnwy2aacfaGaf8hdW3Kbauaaaaa@4199@\\M″
 MathType@MTEF@5@5@+=feaafiart1ev1aaatCvAUfKttLearuWrP9MDH5MBPbIqV92AaeXatLxBI9gBamrtHrhAL1wy0L2yHvtyaeHbnfgDOvwBHrxAJfwnaebbnrfifHhDYfgasaacH8akY=wiFfYdH8Gipec8Eeeu0xXdbba9frFj0=OqFfea0dXdd9vqai=hGuQ8kuc9pgc9s8qqaq=dirpe0xb9q8qiLsFr0=vr0=vr0dc8meaabaqaciaacaGaaeqabaWaaeGaeaaakeaat0uy0HwzTfgDPnwy2aqeh0uy0HwzTfgDPnwy2aacfaGaf8hdW3Kbayaaaaa@419A@|| ≤ ||M′
 MathType@MTEF@5@5@+=feaafiart1ev1aaatCvAUfKttLearuWrP9MDH5MBPbIqV92AaeXatLxBI9gBamrtHrhAL1wy0L2yHvtyaeHbnfgDOvwBHrxAJfwnaebbnrfifHhDYfgasaacH8akY=wiFfYdH8Gipec8Eeeu0xXdbba9frFj0=OqFfea0dXdd9vqai=hGuQ8kuc9pgc9s8qqaq=dirpe0xb9q8qiLsFr0=vr0=vr0dc8meaabaqaciaacaGaaeqabaWaaeGaeaaakeaat0uy0HwzTfgDPnwy2aqeh0uy0HwzTfgDPnwy2aacfaGaf8hdW3Kbauaaaaa@4199@\M″
 MathType@MTEF@5@5@+=feaafiart1ev1aaatCvAUfKttLearuWrP9MDH5MBPbIqV92AaeXatLxBI9gBamrtHrhAL1wy0L2yHvtyaeHbnfgDOvwBHrxAJfwnaebbnrfifHhDYfgasaacH8akY=wiFfYdH8Gipec8Eeeu0xXdbba9frFj0=OqFfea0dXdd9vqai=hGuQ8kuc9pgc9s8qqaq=dirpe0xb9q8qiLsFr0=vr0=vr0dc8meaabaqaciaacaGaaeqabaWaaeGaeaaakeaat0uy0HwzTfgDPnwy2aqeh0uy0HwzTfgDPnwy2aacfaGaf8hdW3Kbayaaaaa@419A@||.

#### Symmetric difference

The *symmetric difference *M
 MathType@MTEF@5@5@+=feaafiart1ev1aaatCvAUfKttLearuWrP9MDH5MBPbIqV92AaeXatLxBI9gBamrtHrhAL1wy0L2yHvtyaeHbnfgDOvwBHrxAJfwnaebbnrfifHhDYfgasaacH8akY=wiFfYdH8Gipec8Eeeu0xXdbba9frFj0=OqFfea0dXdd9vqai=hGuQ8kuc9pgc9s8qqaq=dirpe0xb9q8qiLsFr0=vr0=vr0dc8meaabaqaciaacaGaaeqabaWaaeGaeaaakeaat0uy0HwzTfgDPnwy2aqeh0uy0HwzTfgDPnwy2aacfaGae8hdW3eaaa@418D@ = M′
 MathType@MTEF@5@5@+=feaafiart1ev1aaatCvAUfKttLearuWrP9MDH5MBPbIqV92AaeXatLxBI9gBamrtHrhAL1wy0L2yHvtyaeHbnfgDOvwBHrxAJfwnaebbnrfifHhDYfgasaacH8akY=wiFfYdH8Gipec8Eeeu0xXdbba9frFj0=OqFfea0dXdd9vqai=hGuQ8kuc9pgc9s8qqaq=dirpe0xb9q8qiLsFr0=vr0=vr0dc8meaabaqaciaacaGaaeqabaWaaeGaeaaakeaat0uy0HwzTfgDPnwy2aqeh0uy0HwzTfgDPnwy2aacfaGaf8hdW3Kbauaaaaa@4199@ △ M″
 MathType@MTEF@5@5@+=feaafiart1ev1aaatCvAUfKttLearuWrP9MDH5MBPbIqV92AaeXatLxBI9gBamrtHrhAL1wy0L2yHvtyaeHbnfgDOvwBHrxAJfwnaebbnrfifHhDYfgasaacH8akY=wiFfYdH8Gipec8Eeeu0xXdbba9frFj0=OqFfea0dXdd9vqai=hGuQ8kuc9pgc9s8qqaq=dirpe0xb9q8qiLsFr0=vr0=vr0dc8meaabaqaciaacaGaaeqabaWaaeGaeaaakeaat0uy0HwzTfgDPnwy2aqeh0uy0HwzTfgDPnwy2aacfaGaf8hdW3Kbayaaaaa@419A@ is defined as the clean network M
 MathType@MTEF@5@5@+=feaafiart1ev1aaatCvAUfKttLearuWrP9MDH5MBPbIqV92AaeXatLxBI9gBamrtHrhAL1wy0L2yHvtyaeHbnfgDOvwBHrxAJfwnaebbnrfifHhDYfgasaacH8akY=wiFfYdH8Gipec8Eeeu0xXdbba9frFj0=OqFfea0dXdd9vqai=hGuQ8kuc9pgc9s8qqaq=dirpe0xb9q8qiLsFr0=vr0=vr0dc8meaabaqaciaacaGaaeqabaWaaeGaeaaakeaat0uy0HwzTfgDPnwy2aqeh0uy0HwzTfgDPnwy2aacfaGae8hdW3eaaa@418D@ = (M′
 MathType@MTEF@5@5@+=feaafiart1ev1aaatCvAUfKttLearuWrP9MDH5MBPbIqV92AaeXatLxBI9gBamrtHrhAL1wy0L2yHvtyaeHbnfgDOvwBHrxAJfwnaebbnrfifHhDYfgasaacH8akY=wiFfYdH8Gipec8Eeeu0xXdbba9frFj0=OqFfea0dXdd9vqai=hGuQ8kuc9pgc9s8qqaq=dirpe0xb9q8qiLsFr0=vr0=vr0dc8meaabaqaciaacaGaaeqabaWaaeGaeaaakeaat0uy0HwzTfgDPnwy2aqeh0uy0HwzTfgDPnwy2aacfaGaf8hdW3Kbauaaaaa@4199@ ∪ M″
 MathType@MTEF@5@5@+=feaafiart1ev1aaatCvAUfKttLearuWrP9MDH5MBPbIqV92AaeXatLxBI9gBamrtHrhAL1wy0L2yHvtyaeHbnfgDOvwBHrxAJfwnaebbnrfifHhDYfgasaacH8akY=wiFfYdH8Gipec8Eeeu0xXdbba9frFj0=OqFfea0dXdd9vqai=hGuQ8kuc9pgc9s8qqaq=dirpe0xb9q8qiLsFr0=vr0=vr0dc8meaabaqaciaacaGaaeqabaWaaeGaeaaakeaat0uy0HwzTfgDPnwy2aqeh0uy0HwzTfgDPnwy2aacfaGaf8hdW3Kbayaaaaa@419A@)\(M′
 MathType@MTEF@5@5@+=feaafiart1ev1aaatCvAUfKttLearuWrP9MDH5MBPbIqV92AaeXatLxBI9gBamrtHrhAL1wy0L2yHvtyaeHbnfgDOvwBHrxAJfwnaebbnrfifHhDYfgasaacH8akY=wiFfYdH8Gipec8Eeeu0xXdbba9frFj0=OqFfea0dXdd9vqai=hGuQ8kuc9pgc9s8qqaq=dirpe0xb9q8qiLsFr0=vr0=vr0dc8meaabaqaciaacaGaaeqabaWaaeGaeaaakeaat0uy0HwzTfgDPnwy2aqeh0uy0HwzTfgDPnwy2aacfaGaf8hdW3Kbauaaaaa@4199@ ∩ M″
 MathType@MTEF@5@5@+=feaafiart1ev1aaatCvAUfKttLearuWrP9MDH5MBPbIqV92AaeXatLxBI9gBamrtHrhAL1wy0L2yHvtyaeHbnfgDOvwBHrxAJfwnaebbnrfifHhDYfgasaacH8akY=wiFfYdH8Gipec8Eeeu0xXdbba9frFj0=OqFfea0dXdd9vqai=hGuQ8kuc9pgc9s8qqaq=dirpe0xb9q8qiLsFr0=vr0=vr0dc8meaabaqaciaacaGaaeqabaWaaeGaeaaakeaat0uy0HwzTfgDPnwy2aqeh0uy0HwzTfgDPnwy2aacfaGaf8hdW3Kbayaaaaa@419A@).

#### Strict symmetric difference

The *strict symmetric difference *M
 MathType@MTEF@5@5@+=feaafiart1ev1aaatCvAUfKttLearuWrP9MDH5MBPbIqV92AaeXatLxBI9gBamrtHrhAL1wy0L2yHvtyaeHbnfgDOvwBHrxAJfwnaebbnrfifHhDYfgasaacH8akY=wiFfYdH8Gipec8Eeeu0xXdbba9frFj0=OqFfea0dXdd9vqai=hGuQ8kuc9pgc9s8qqaq=dirpe0xb9q8qiLsFr0=vr0=vr0dc8meaabaqaciaacaGaaeqabaWaaeGaeaaakeaat0uy0HwzTfgDPnwy2aqeh0uy0HwzTfgDPnwy2aacfaGae8hdW3eaaa@418D@ = M′
 MathType@MTEF@5@5@+=feaafiart1ev1aaatCvAUfKttLearuWrP9MDH5MBPbIqV92AaeXatLxBI9gBamrtHrhAL1wy0L2yHvtyaeHbnfgDOvwBHrxAJfwnaebbnrfifHhDYfgasaacH8akY=wiFfYdH8Gipec8Eeeu0xXdbba9frFj0=OqFfea0dXdd9vqai=hGuQ8kuc9pgc9s8qqaq=dirpe0xb9q8qiLsFr0=vr0=vr0dc8meaabaqaciaacaGaaeqabaWaaeGaeaaakeaat0uy0HwzTfgDPnwy2aqeh0uy0HwzTfgDPnwy2aacfaGaf8hdW3Kbauaaaaa@4199@ ◇ M″
 MathType@MTEF@5@5@+=feaafiart1ev1aaatCvAUfKttLearuWrP9MDH5MBPbIqV92AaeXatLxBI9gBamrtHrhAL1wy0L2yHvtyaeHbnfgDOvwBHrxAJfwnaebbnrfifHhDYfgasaacH8akY=wiFfYdH8Gipec8Eeeu0xXdbba9frFj0=OqFfea0dXdd9vqai=hGuQ8kuc9pgc9s8qqaq=dirpe0xb9q8qiLsFr0=vr0=vr0dc8meaabaqaciaacaGaaeqabaWaaeGaeaaakeaat0uy0HwzTfgDPnwy2aqeh0uy0HwzTfgDPnwy2aacfaGaf8hdW3Kbayaaaaa@419A@ is M
 MathType@MTEF@5@5@+=feaafiart1ev1aaatCvAUfKttLearuWrP9MDH5MBPbIqV92AaeXatLxBI9gBamrtHrhAL1wy0L2yHvtyaeHbnfgDOvwBHrxAJfwnaebbnrfifHhDYfgasaacH8akY=wiFfYdH8Gipec8Eeeu0xXdbba9frFj0=OqFfea0dXdd9vqai=hGuQ8kuc9pgc9s8qqaq=dirpe0xb9q8qiLsFr0=vr0=vr0dc8meaabaqaciaacaGaaeqabaWaaeGaeaaakeaat0uy0HwzTfgDPnwy2aqeh0uy0HwzTfgDPnwy2aacfaGae8hdW3eaaa@418D@ = (M′
 MathType@MTEF@5@5@+=feaafiart1ev1aaatCvAUfKttLearuWrP9MDH5MBPbIqV92AaeXatLxBI9gBamrtHrhAL1wy0L2yHvtyaeHbnfgDOvwBHrxAJfwnaebbnrfifHhDYfgasaacH8akY=wiFfYdH8Gipec8Eeeu0xXdbba9frFj0=OqFfea0dXdd9vqai=hGuQ8kuc9pgc9s8qqaq=dirpe0xb9q8qiLsFr0=vr0=vr0dc8meaabaqaciaacaGaaeqabaWaaeGaeaaakeaat0uy0HwzTfgDPnwy2aqeh0uy0HwzTfgDPnwy2aacfaGaf8hdW3Kbauaaaaa@4199@ ∪ M″
 MathType@MTEF@5@5@+=feaafiart1ev1aaatCvAUfKttLearuWrP9MDH5MBPbIqV92AaeXatLxBI9gBamrtHrhAL1wy0L2yHvtyaeHbnfgDOvwBHrxAJfwnaebbnrfifHhDYfgasaacH8akY=wiFfYdH8Gipec8Eeeu0xXdbba9frFj0=OqFfea0dXdd9vqai=hGuQ8kuc9pgc9s8qqaq=dirpe0xb9q8qiLsFr0=vr0=vr0dc8meaabaqaciaacaGaaeqabaWaaeGaeaaakeaat0uy0HwzTfgDPnwy2aqeh0uy0HwzTfgDPnwy2aacfaGaf8hdW3Kbayaaaaa@419A@)\\(M′
 MathType@MTEF@5@5@+=feaafiart1ev1aaatCvAUfKttLearuWrP9MDH5MBPbIqV92AaeXatLxBI9gBamrtHrhAL1wy0L2yHvtyaeHbnfgDOvwBHrxAJfwnaebbnrfifHhDYfgasaacH8akY=wiFfYdH8Gipec8Eeeu0xXdbba9frFj0=OqFfea0dXdd9vqai=hGuQ8kuc9pgc9s8qqaq=dirpe0xb9q8qiLsFr0=vr0=vr0dc8meaabaqaciaacaGaaeqabaWaaeGaeaaakeaat0uy0HwzTfgDPnwy2aqeh0uy0HwzTfgDPnwy2aacfaGaf8hdW3Kbauaaaaa@4199@ ∩ M″
 MathType@MTEF@5@5@+=feaafiart1ev1aaatCvAUfKttLearuWrP9MDH5MBPbIqV92AaeXatLxBI9gBamrtHrhAL1wy0L2yHvtyaeHbnfgDOvwBHrxAJfwnaebbnrfifHhDYfgasaacH8akY=wiFfYdH8Gipec8Eeeu0xXdbba9frFj0=OqFfea0dXdd9vqai=hGuQ8kuc9pgc9s8qqaq=dirpe0xb9q8qiLsFr0=vr0=vr0dc8meaabaqaciaacaGaaeqabaWaaeGaeaaakeaat0uy0HwzTfgDPnwy2aqeh0uy0HwzTfgDPnwy2aacfaGaf8hdW3Kbayaaaaa@419A@).

The Vienna Reaction Network Library Vienna-RNL implements these basic set-theoretic operations on chemical reaction networks. It is available under the GNU Public License from [32] and as additional file: 1. The library is written in platform independent ANSI C and provides basic data structures for chemical reactions and their networks, IO routines for reading and writing and various formats, as well as set operations such as the union, intersection, or difference of two chemical reaction networks. It is intended for the use in conjunction with the user's own C programs or PERL scripts.

The library contains IO-Routines for reading SBML [23], an XML based dialect for the standardized representation of systems biology models, writing of SBML is currently being implemented. The capability of reading and writing SBML will make the functionality of the Vienna Reaction Network Library accessible to about 80 other software systems which support SBML [33].

### Phylogenies from networks

Datasets were retrieved from the KEGG database on metabolic networks [2], which holds genomic and network data of about 20 Archaea, 200 Bacteria and 20 Eucarya, where in particular the data of many Eukaryotes are incomplete. In a preparatory step we decomposed the individual KEGG-pathways into their chemical reactions and combined these to a complete network for each organism.

The simplest approach to inferring phylogenetic relationships from metabolic networks is to use a distance measure *d *on the set of reaction networks. We use here

d(M′,M″)=‖M′△M″‖‖M′‖+‖M″‖−‖M′∩M″‖=‖M′△M″‖‖M′∪M″‖     (7)
 MathType@MTEF@5@5@+=feaafiart1ev1aaatCvAUfKttLearuWrP9MDH5MBPbIqV92AaeXatLxBI9gBamrtHrhAL1wy0L2yHvtyaeHbnfgDOvwBHrxAJfwnaebbnrfifHhDYfgasaacH8akY=wiFfYdH8Gipec8Eeeu0xXdbba9frFj0=OqFfea0dXdd9vqai=hGuQ8kuc9pgc9s8qqaq=dirpe0xb9q8qiLsFr0=vr0=vr0dc8meaabaqaciaacaGaaeqabaWaaeGaeaaakeaacqWGKbazcqGGOaakt0uy0HwzTfgDPnwy2aqeh0uy0HwzTfgDPnwy2aacfaGaf8hdW3KbauaacqGGSaalcuWFmaFtgaGbaiabcMcaPiabg2da9maalaaabaWaauWaaeaacuWFmaFtgaqbamrr1ngBPrwtHrhAYaqeiuuDJXwAKbstHrhAGq1DVbacgaGae43SLeIaf8hdW3KbayaaaiaawMa7caGLkWoaaeaadaqbdaqaaiqb=Xa8nzaafaaacaGLjWUaayPcSdGaey4kaSYaauWaaeaacuWFmaFtgaGbaaGaayzcSlaawQa7aiabgkHiTmaafmaabaGaf8hdW3KbauaacqGHPiYXcuWFmaFtgaGbaaGaayzcSlaawQa7aaaacqGH9aqpdaWcaaqaamaafmaabaGaf8hdW3KbauaacqGFZwsicuWFmaFtgaGbaaGaayzcSlaawQa7aaqaamaafmaabaGaf8hdW3KbauaacqGHQicYcuWFmaFtgaGbaaGaayzcSlaawQa7aaaacaWLjaGaaCzcamaabmaabaGaeG4naCdacaGLOaGaayzkaaaaaa@846C@

Alternatively, the strict symmetric difference M′
 MathType@MTEF@5@5@+=feaafiart1ev1aaatCvAUfKttLearuWrP9MDH5MBPbIqV92AaeXatLxBI9gBamrtHrhAL1wy0L2yHvtyaeHbnfgDOvwBHrxAJfwnaebbnrfifHhDYfgasaacH8akY=wiFfYdH8Gipec8Eeeu0xXdbba9frFj0=OqFfea0dXdd9vqai=hGuQ8kuc9pgc9s8qqaq=dirpe0xb9q8qiLsFr0=vr0=vr0dc8meaabaqaciaacaGaaeqabaWaaeGaeaaakeaat0uy0HwzTfgDPnwy2aqeh0uy0HwzTfgDPnwy2aacfaGaf8hdW3Kbauaaaaa@4199@ ◇ M″
 MathType@MTEF@5@5@+=feaafiart1ev1aaatCvAUfKttLearuWrP9MDH5MBPbIqV92AaeXatLxBI9gBamrtHrhAL1wy0L2yHvtyaeHbnfgDOvwBHrxAJfwnaebbnrfifHhDYfgasaacH8akY=wiFfYdH8Gipec8Eeeu0xXdbba9frFj0=OqFfea0dXdd9vqai=hGuQ8kuc9pgc9s8qqaq=dirpe0xb9q8qiLsFr0=vr0=vr0dc8meaabaqaciaacaGaaeqabaWaaeGaeaaakeaat0uy0HwzTfgDPnwy2aqeh0uy0HwzTfgDPnwy2aacfaGaf8hdW3Kbayaaaaa@419A@could be used to define a difference measure. Furthermore, other normalizations of the difference measure could be used. By calculating tree distances between the 16S rRNA phylogenies and the network phylogenies, using the treedist program from the phylip package [24], we have observed, however, that equ.(7) performs best with respect to reproducing trusted 16S RNA phylogenies. Tree distances between the 16S rRNA phylogeny and the network phylogeny calculated with the symmetric difference are 2 and 0.023, using symmetric difference and branch score distance measure, respectively. Compared with tree distances between the 16S rRNA phylogeny and the network phylogeny utilizing the strict symmetric difference yields a much larger tree distance of 10 and 0.093, respectively.

Distance-based network phylogenies are computed using the Fitch algorithm [25] implemented in the phylip package as well as using the splits-decomposition algorithm from the SplitsTree package [26].

**Figure 2:**
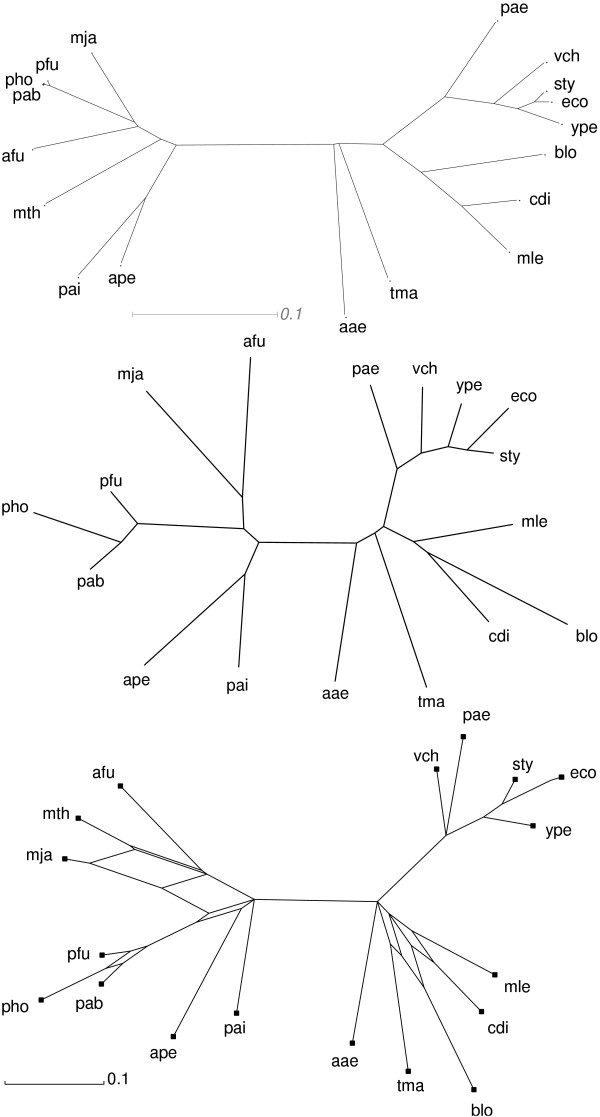
Unrooted phylogenies. (top) Maximum parsimony tree of 16S rRNA sequences. (center) Phy-logenetic tree calculated from metabolic network data using the Fitch algorithm for distance matrices. (bottom) Phylogenetic tree calculated from metabolic network data using Splits decomposition with the Fitch-Margoliash power 2 fit for distance matrices. Species abbreviations are collected in Table 1.

An example comprising a selection of bacterial and archaeal metabolic networks is shown in Fig. 2, see Table 1 for the list of species used. The phylogeny inferred from the metabolic networks conforms almost perfectly with the maximum parsimony tree computed from the 16S rRNA sequences of the same organisms. The rRNA sequences were aligned using clustalx. The minor discrepancies are due to poorly resolved nodes as can be seen in the split-decomposition network below. The congruence of rRNA trees and network-based phylogenies demonstrates that the structure of metabolic networks encodes phylogenetic information. Metabolic networks thus constitute a source of phylogenetic signal that is completely independent of sequence information.

**Table 1:** Metabolic networks used in this study.

Domain			Species	KEGG Id	Genomic Sequence
Bacteria	Proteobacteria	Gamma	Escherichia coli K-12 MG1655	eco	U00096
			Buchnera aphidicola	buc	BA_000003
			Salmonella typhi CT18	sty	NC_003198
			Yersinia pestis CO92	ype	NC_003143
			Vibrio cholorae	vch	NC_002505
			Pseudomonas aeruginosa	pae	NC_002516
		
		Rickettsiales	Rickettsia prowazekii	rpr	NC_000963
			Wolbachia endosymbiont	wol	NC_002978
	
	Firmicutes	Mollicutes	Mycoplasmae genitalium	mge	L43967
			Mycoplasmae pneumoniae	mpn	NC_000912
			Ureaplasmae urealyticum	uur	NC_002162
	
	Spirochaetes		Borrelia burgdorferi	bbu	AE000783
			Treponema pallidum	tpa	NC_000919
			Treponema denticola	tde	NC_002967
	
	Actinobacteria		Mycobacterium leprae	mle	NC_002677
			Bifidobacterium longum	blo	NC_004307
			Corynebacterium diphtheriae	cdi	NC_002935
	
	Hyperthermophilic bacteria		Aquifex aeolicus	aae	AE000657
			Thermotoga maritima	tma	AE000512

Archaea	Euryarchaeota		Methanocaldococcus jannaschii	mja	NC_000909
			Methanothermobacter thermoautotrophicus	mth	NC_000916
			Archaeoglobus fulgidus	afu	NC_000917
			Pyrococcus horikoshii	pho	BA000001
			Pyrococcus abyssi	pab	NC_000865
			Pyrococcus furiosus	pfu	NC_003413
	
	Crenarchaeota		Aeropyrum pernix	ape	BA000002
			Pyrobaculum aerophilum	pai	NC_003364

The use of distance measures reduces the available information on the network structure already in the first step. We therefore complement distance based phylogenetic analysis with parsimony methods using reaction content: For a given set of organisms, we first calculated the union U
 MathType@MTEF@5@5@+=feaafiart1ev1aaatCvAUfeBSjuyZL2yd9gzLbvyNv2CaerbwvMCKfMBHbqedmvETj2BSbWenfgDOvwBHrxAJfwnHbqeg0uy0HwzTfgDPnwy1aqee0evGueE0jxyaibaieYdOi=BH8vipeYdI8qiW7rqqrFfpeea0xe9Lq=Jc9vqaqpepm0xbbG8FasPYRqj0=yi0lXdbba9pGe9qqFf0dXdHuk9fr=xfr=xfrpiWZqaaeaabiGaaiaacaqabeaadaqacqaaaOqaamrtHrhAL1wy0L2yHzdarCqtHrhAL1wy0L2yHzdaiuaacqWFuaFvaaa@44B3@ of their metabolic networks. For each organism we then constructed a reaction profile reflecting presence or absence of each reaction in U
 MathType@MTEF@5@5@+=feaafiart1ev1aaatCvAUfeBSjuyZL2yd9gzLbvyNv2CaerbwvMCKfMBHbqedmvETj2BSbWenfgDOvwBHrxAJfwnHbqeg0uy0HwzTfgDPnwy1aqee0evGueE0jxyaibaieYdOi=BH8vipeYdI8qiW7rqqrFfpeea0xe9Lq=Jc9vqaqpepm0xbbG8FasPYRqj0=yi0lXdbba9pGe9qqFf0dXdHuk9fr=xfr=xfrpiWZqaaeaabiGaaiaacaqabeaadaqacqaaaOqaamrtHrhAL1wy0L2yHzdarCqtHrhAL1wy0L2yHzdaiuaacqWFuaFvaaa@44B3@ in the metabolic network of the respective organism. This approach thus is reduced to reconstructing phylogenies from character-tables that represent the presence/absence of particular reactions in the reaction network. It should be noted that this is similar, but not quite the same, as using the presence or absence of orthologous enzymes (see e.g. [3-8]). The main difference is that the network based approach tolerates functional replacements that may occur, e.g., through horizontal gene transfer [9].

### Metabolic innovations

The algebraic approach to metabolic network evolution can also be used in a straightforward way to trace the history of metabolic innovations. To this end, consider a (trusted) unrooted phylogenetic tree *T *in which each leaf of *T *is labeled with the metabolic network M
 MathType@MTEF@5@5@+=feaafiart1ev1aaatCvAUfKttLearuWrP9MDH5MBPbIqV92AaeXatLxBI9gBamrtHrhAL1wy0L2yHvtyaeHbnfgDOvwBHrxAJfwnaebbnrfifHhDYfgasaacH8akY=wiFfYdH8Gipec8Eeeu0xXdbba9frFj0=OqFfea0dXdd9vqai=hGuQ8kuc9pgc9s8qqaq=dirpe0xb9q8qiLsFr0=vr0=vr0dc8meaabaqaciaacaGaaeqabaWaaeGaeaaakeaat0uy0HwzTfgDPnwy2aqeh0uy0HwzTfgDPnwy2aacfaGae8hdW3eaaa@418D@_*k *_of the corresponding taxon *k*. Each edge *e *of *T *defines a split, i.e., a bipartition *σ*_*e *_= {*U*_*e*_, _*e*_} of the set of taxa. Here we regard splits as directed. Note that mathematically we can define innovations at each split in both directions. One of the two subsets *U *or , however, contains the ancestral state, hence only one direction makes biological sense: this is the one where the ancestral state (root of the tree) is located in the sub-set . This knowledge has to be provided externally.

Consider an (arbitrary) directed split *σ *= (*U*, ) on the given set of taxa, i.e., a pair of sets of taxa (*U*, ) such that *U * θ,  θ, and *U *∩  = θ. We define the differential metabolic network



The network D
 MathType@MTEF@5@5@+=feaafiart1ev1aaatCvAUfKttLearuWrP9MDH5MBPbIqV92AaeXatLxBI9gBamrtHrhAL1wy0L2yHvtyaeHbnfgDOvwBHrxAJfwnaebbnrfifHhDYfgasaacH8akY=wiFfYdH8Gipec8Eeeu0xXdbba9frFj0=OqFfea0dXdd9vqai=hGuQ8kuc9pgc9s8qqaq=dirpe0xb9q8qiLsFr0=vr0=vr0dc8meaabaqaciaacaGaaeqabaWaaeGaeaaakeaaimaacqWFdepraaa@3827@(*σ*) describes the *metabolic innovations *in *U *relative to the "background" U¯
 MathType@MTEF@5@5@+=feaafiart1ev1aaatCvAUfKttLearuWrP9MDH5MBPbIqV92AaeXatLxBI9gBaebbnrfifHhDYfgasaacH8akY=wiFfYdH8Gipec8Eeeu0xXdbba9frFj0=OqFfea0dXdd9vqai=hGuQ8kuc9pgc9s8qqaq=dirpe0xb9q8qiLsFr0=vr0=vr0dc8meaabaqaciaacaGaaeqabaqabeGadaaakeaacuWGvbqvgaqeaaaa@2DF7@.

As discussed in the previous section, network phylogenies are rather sensitive with respect to life-style and environmental constraints. The organisms whose metabolic networks have been utilized to compute the 16s rRNA tree shown in Fig. 2 are able to freely live in the environment with a reasonably large capacity for adaptation.

**Figure 3:**
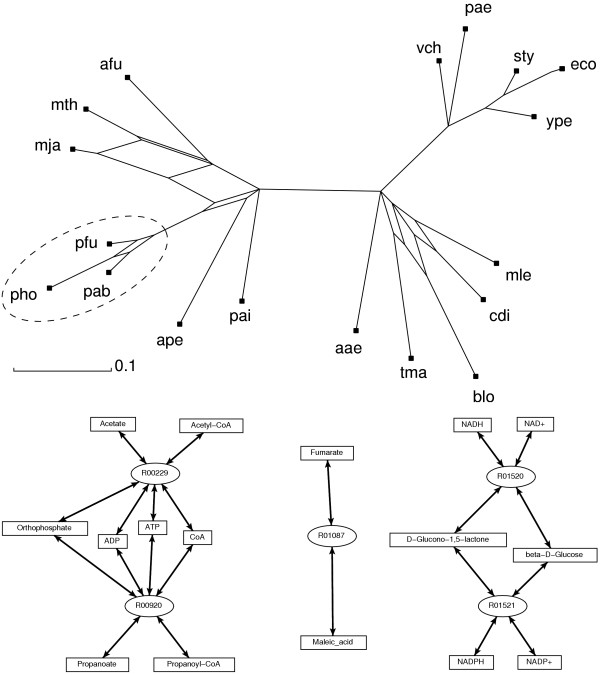
(top) The *Pyrococcus spp*. clade has been selected (dashed oval) for differential network analysis. (bottom) Differential metabolic network. Numbers in the ovals refer to reaction ids in the KEGG database.

As a first example we analyzed the unique metabolic network from the *Pyrococcus *genus. Figure 3 shows the network phylogeny from Fig. 2 with the *Pyrococcus spp*. clade highlighted. The resulting differential network indicates reactions present in *Pyrococcus spp*. but absent in all other organisms of the phylogeny (Figure 3). For example, reaction *R*01087 is catalyzed by Maleate cis-trans-isomerase which is utilized in maleate assimilating and high-temperature bacteria. A second sub-network involving both ADP-forming acetate and propanoate CoA ligases is potentially used in the organisms to convert between acetate and propanoate and their corresponding CoA forms.

**Figure 4:**
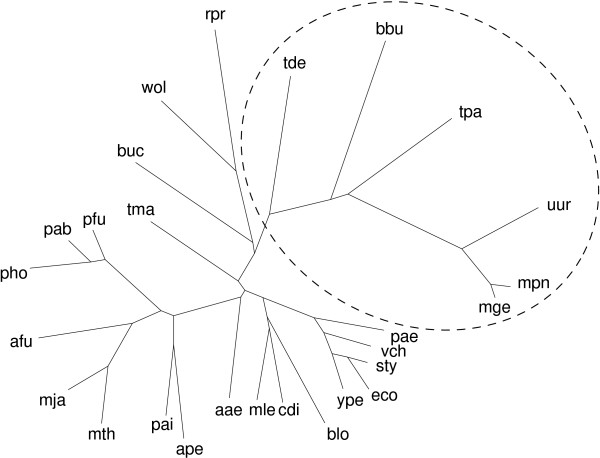
Unrooted network phylogeny using PHYLIP with the Fitch-Margoliash algorithm. A set of obligatory intracellular pathogens has been selected (dashed oval) for differential network analysis (see text).

**Figure 5:**
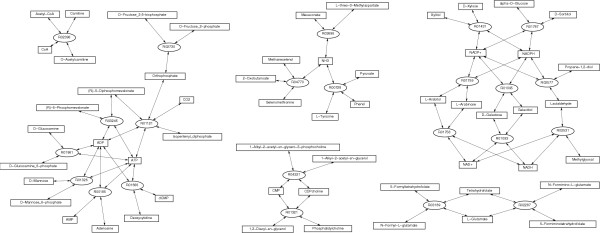
Differential network corresponding the split shown in Figure 4. These reactions are specializations of the intracellular parasites.

As a second example we analyzed our set of reference organism (Figure 2) with obligatory intracellular pathogens. Figure 4 shows the phylogeny with the selected pathogens (dashed oval). Interestingly, *Mollicutes*, such as *Mycoplasmae *and *Spirochaetes*, such as *Treponema *are grouped together. They all possess a minimal gene-set, and thus a highly optimized and host-dependent metabolic network. Surprisingly, this set of organisms has specific reactions that are absent in the remaining organisms of the phylogeny. Figure 5 shows the corresponding differential network which consists of five sub-network. The two largest networks involve sugar-conversions and parts of glycolysis. Smaller networks correspond to formylation of tetrahydrofolate as well as cholin and carnitine pathways.

## Discussion

The Vienna Reaction Network Library introduced above treats chemical reaction networks, and metabolic networks in particular, as directed hypergraphs. A framework borrowed from set algebra provides natural definitions of unions, intersections, and differences that can be used to compare the metabolic networks of different organisms. We have demonstrated that metabolic networks convey phylogenetic information and can indeed be used to infer phylogenetic relationships of free-living organisms in a way that is similar to gene-content based approaches. In contrast to the latter, however, metabolic network based phylogenies are less sensitive to the effects of functional replacement, e.g., through horizontal gene transfer.

Differences of metabolic networks among subtrees of a trusted phylogeny, or more generally, along any split of interest in a set of organisms can be computed directly, making it easy to study metabolic innovations in particular clades. A first application of our network phylogeny analysis involved three members of the *Pyrococcus spp*. clade. The metabolic reactions resulting from the split between the *Pyrococci *and the remaining organisms involve the maleate cis-trans-isomerase reaction, ADP-forming acetate and propanoate CoA ligase reactions as well as beta-D-Glucose:NAD(P)^+^ 1-oxoreductase.

Our second example considers a class of intra-cellular pathogens that includes *Mycoplasmae, Ureaplasmae*, and *Spirochete*. Their restricted repertoire of metabolic reactions reflect the specialized life-style. Many metabolic pathways are not required in such a rich environment and have been lost in the course of evolution. On the other hand, constructing a network phylogeny including these microbes, we observe metabolic reactions assembling an unconnected network that is present in this set of intracellular pathogens and absent in the remaining organisms. Such reactions include phosphorilization and conversions of sugars and derivates, deaminating lyase reactions, and reactions involving carnitine, choline and tetrahydrofolate.

At present, metabolic network data are compiled by a multitude of methods, and at least in part are constructed by genomic similarity with other organisms. Strictly speaking, therefore, we cannot view metabolic network data such as those complied in the KEGG database as independent from genomic data. With the recent advances of experimental techniques in metabolomics (see e.g. [27-29]), however, the situation is rapidly improving.

## Conclusion

Our comparative approach to metabolic network analysis, which focuses on individual reactions rather than on aggregate features such as pathways, simplifies the identification of metabolic innovations and, in particular, facilitates the recognition of organisms as potential biological threat agents based on their metabolic repertoire. Furthermore, the ability to easily identify differences in metabolic capacity between pathogens should be useful for devising a refined classification of pathogenicity based on metabolic capabilities.

In this contribution we have restricted ourselves to unweighted networks. Distance measures between networks, however, could be refined by attaching weights to both vertices and (hyper-)edges without requiring significant algorithmic changes. These could reflect, e.g., how essential a reaction or a metabolite is for each organism. With the increasing amount and accuracy of available data it might also be feasible to devise a stochastic model of the evolution of metabolic networks, which could then be turned into a scoring scheme for a generalized version of (local) graph alignment along the lines of [30].

## Authors' contributions

The idea on a metabolic network algebra has been conceived by ILH and PFS during a visit at the Santa Fe Institute. The implementation was substantially improved by CF. CVF developed the distance measurements and applications to microbial metabolic networks. All authors collaborated closely in writing the manuscript.

## Supplementary Material

Additional file 1 The Vienna Reaction Network Library Vienna-RNL implements these basic set-theoretic operations on chemical reaction networks. It is available under the GNU Public License from [32] Click here for file
